# Protocol for a randomized clinical trial exploring the effect of antimicrobial agents on the penile microbiota, immunology and HIV susceptibility of Ugandan men

**DOI:** 10.1186/s13063-019-3545-7

**Published:** 2019-07-19

**Authors:** Ronald M. Galiwango, Bernard Bagaya, Juliet Mpendo, Vineet Joag, Brenda Okech, Annet Nanvubya, Ali Ssetaala, Moses Muwanga, Rupert Kaul

**Affiliations:** 1 Department of Immunology, University of Toronto, St. George Campus Medical Sciences Building #6356 1 King’s College Circle, Toronto, ON M5S 1A8 Canada; 2 Department of Medicine, University of Toronto, Toronto, ON Canada; 3 HIV Vaccine Program, Uganda Virus Research Institute–International AIDS Vaccine Initiative, Entebbe, Uganda; 4 Department of Microbiology, Makerere University College of Health Sciences, Kampala, Uganda; 5 Entebbe General Hospital, Entebbe, Uganda; 6 Department of Medicine, University Health Network, Toronto, ON Canada

**Keywords:** HIV transmission, Penile microbiome, Clinical trial, Antimicrobials, Uganda

## Abstract

**Background:**

The foreskin is the main site of HIV acquisition in a heterosexual uncircumcised man, but many men in endemic countries are reluctant to undergo penile circumcision (PC). Observational studies suggest that proinflammatory anaerobic bacteria are enriched on the uncircumcised penis, where they may enhance HIV susceptibility through increased foreskin inflammatory cytokines and the recruitment of HIV-susceptible CD4^+^ target cells. This trial will examine the impact of systemic and topical antimicrobials on ex vivo foreskin HIV susceptibility.

**Methods/design:**

This randomized, open-label clinical trial will randomize 125 HIV-negative Ugandan men requesting voluntary PC to one of five arms (*n* = 25 each). The control group will receive immediate PC, while the four intervention groups will defer PC for 1 month and be provided in the interim with either oral tinidazole, penile topical metronidazole, topical clindamycin, or topical hydrogen peroxide. The impact of these interventions on HIV entry into foreskin-derived CD4^+^ T cells will be quantified ex vivo at the time of PC using a clade A, R5 tropic HIV pseudovirus assay (primary endpoint); secondary endpoints include the impact of antimicrobials on immune parameters and the microbiota of the participant’s penis and of the vagina of their female partner (if applicable), assessed by multiplex enzyme-linked immunosorbent assay and 16S rRNA sequencing.

**Discussion:**

There is a critical need to develop acceptable, simple, and effective means of HIV prevention in men unwilling to undergo PC. This trial will provide insight into the causative role of the foreskin microbiota on HIV susceptibility, and the impact of simple microbiota-focused clinical interventions. This may pave the way for future clinical trials using low-cost, nonsurgical intervention(s) to reduce HIV risk in uncircumcised heterosexual men.

**Trial registration:**

ClinicalTrials.gov, NCT03412071. Retrospectively registered on 26 January 2018.

**Electronic supplementary material:**

The online version of this article (10.1186/s13063-019-3545-7) contains supplementary material, which is available to authorized users.

## Background

The provision of early and effective antiretroviral therapy for a person infected with HIV reduces the risk of transmission to their partner to negligible levels, and consistent use by an uninfected person of pre-exposure prophylaxis (PrEP) reduces their risk of HIV acquisition by 90–95% [1, 2]. However, the global rate of new HIV infections outstrips current treatment capacity, and the ability and political will to fund expansion of PrEP programs in endemic countries is not clear; furthermore, the emergence of viral resistance in sub-Saharan Africa is a real concern [3, 4]. Safe penile circumcision (PC) reduces HIV susceptibility by almost 60% in heterosexual men and is being actively implemented in 14 priority sub-Saharan African countries where infant circumcision has not been the cultural norm [5–7]. Nonetheless, many eligible men choose not to undergo this surgical procedure, and PC implementation is falling well short of global targets [8, 9]. Based on the key role of the foreskin in HIV acquisition [10], new nonsurgical prevention tools that are acceptable to men who choose to remain uncircumcised could enhance prevention efforts.

## Foreskin inflammation associated with penile anaerobes increases HIV risk

Although the immunopathogenesis of HIV acquisition in the foreskin is poorly understood, observational studies have demonstrated strong immune and microbial associations of HIV acquisition that suggest new avenues for prevention. Specifically, foreskin tissues are enriched for highly HIV-susceptible CD4^+^ T cell subsets, including T helper 17 (Th17) cells and CD4^+^ T cells expressing the HIV coreceptor CCR5 [11], and these highly susceptible CD4^+^ T cell subsets were reduced in the foreskins of HIV-exposed but seronegative men [12]. This suggests that foreskin inflammation and/or immune activation enhances HIV susceptibility and, in keeping with this, prepuce levels of the chemotactic cytokine interleukin-8 (IL-8) were directly associated with an increased risk of HIV acquisition in Ugandan men, and correlated with an increased tissue density of HIV-susceptible CD4^+^ subsets [13]. Interestingly, these immune parameters also strongly correlated with the density of gram-negative anaerobic bacteria, such as *Prevotella* spp., in the foreskin prepuce [13–15], and PC has been previously shown to reduce the prevalence of these penile bacteria [16].

## Hypothesis

Based on these observations, we hypothesize that host immune alterations induced in the uncircumcised penis by gram-negative anaerobic bacteria enhance HIV susceptibility, and that oral and/or topical antimicrobials will reduce foreskin anaerobe load, local tissue inflammation, and therefore lower foreskin susceptibility to HIV.

## Antimicrobial modification of the penile microbiome may reduce HIV risk

Modification of the penile microbiota composition, specifically a reduction in genital anaerobes, might therefore reduce foreskin tissue inflammation and constitute a novel, low-tech avenue for HIV prevention in men at risk of HIV who do not wish to be circumcised [8]. However, it is not known which antimicrobial agents (if any) can optimally modify the penile microbiota, or whether penile microbiota alterations will reduce foreskin inflammation and HIV susceptibility. This proposed clinical trial will examine the effect of microbiota-targeted interventions on foreskin HIV susceptibility. Specifically, male participants will be provided with commonly available, licensed antibacterial agents prior to elective penile circumcision. Genital swabs and tissues collected at the time of PC will then be tested to assess the impact on foreskin microbiota, immune parameters and cellular HIV susceptibility. Therefore, the primary goal of this clinical trial is to translate prior basic science research into low-tech, affordable interventions that can reduce HIV risk in uncircumcised men identifying themselves as heterosexual, and extend our understanding of penile immunology and HIV susceptibility.

## Methods/design

### Study design and site

This study is an open-label, randomized clinical trial assessing the effect of common antibacterials on foreskin HIV susceptibility, immunology, and microbiota composition. The study is being conducted at the Uganda Virus Research Institute (UVRI)–International Aids Vaccine Initiative (IAVI) and Entebbe General Hospital in Entebbe, Uganda.

### Study population

A total of 125 HIV-negative Ugandan men will be enrolled. Eligible men are aged ≥ 18 years seeking voluntary PC at Entebbe General Hospital in Uganda. Men will be invited to participate in the study during the health education and HIV counseling/testing sessions routinely held at the clinic. If men are in a stable heterosexual relationship, their female partners will also be eligible for participation in the study.

### Study treatment groups and randomization

Eligible men (Table 1), who have provided informed, written consent to participate in the trial, will self-randomize, in a computer-generated, open-label fashion to any of the study groups. The study enroller randomly shuffles a pile of sealed, opaque envelopes in the presence of the participant, who then picks a single envelope from the pile. Inside the envelope are details about the participant’s randomization to one of the five study groups (*n* = 25 participants per group), as follows: group 1 is the no treatment (control) group which undergoes immediate PC, as per the normal Entebbe General Hospital protocol; in group 2, participants defer PC for 4 weeks and are provided with tinidazole 2 g by mouth once daily for 2 days (this imidazole class is indicated for treatment of several conditions including amebiasis, giardiasis, trichomoniasis and bacterial vaginosis [17, 18]); in group 3, participants defer PC for 4 weeks and apply topical metronidazole cream 0.75% underneath the foreskin twice daily for 1 week and then twice weekly for the remaining 3 weeks (this topical imidazole is indicated for treatment of bacterial vaginosis [18]); in group 4, participants defer PC for 4 weeks and apply topical clindamycin 2% cream underneath the foreskin twice daily for 1 week and then twice weekly for the remaining 3 weeks (this topical antibiotic is indicated for treatment of bacterial vaginosis [18]); in group 5, participants defer PC for 4 weeks and apply topical hydrogen peroxide 1% gel underneath the foreskin twice daily for 1 week and then twice weekly for the remaining 2 weeks (hydrogen peroxide preparations are indicated for topical treatment of gingivitis/periodontitis [19, 20]). Men in intervention groups 3, 4, and 5 will be reminded about product use twice a week via a telephone call.

**Table 1:** Inclusion and exclusion criteria

Inclusion criteria	
Aged ≥18 years	
Male	
Uncircumcised	
HIV-negative	
Willing to give written informed consent	
Willing and able to answer a short social-behavior questionnaire	
Willing to comply with study protocol requirements including randomization and study drug usage	
Available for planned duration of randomization group	
Exclusion criteria	
HIV-infected	
Already circumcised	
Self-reported or physician-noted genital itching/burning, penile discharge, genital ulceration or other possible sexually transmitted infection symptoms	
Participating in other research studies that might compromise the outcomes of this study.	

### Study outcomes

The broad objectives of this clinical trial are: 1) to quantify the impact of antimicrobial agents on foreskin HIV susceptibility, immunology, and microbiota; and 2) to assess the potential impact of male partner antimicrobial use on the genital immunology and microbiota in their female sexual partner. The primary study endpoint is the percentage HIV pseudovirus entry into foreskin-derived CD4^+^ T cells in the intervention versus control arms, utilizing a validated primary clade A, R5 tropic pseudovirus entry assay [21, 22]. Secondary study endpoints include: 1) foreskin tissue density of HIV-susceptible CD4^+^ T cells; 2) the density and percentage of CD4^+^ T cell subsets (including Th17 cells) in foreskin tissue; 3) the density of Langerhans cells in foreskin tissue based on immunofluorescence microscopy; 4) the presence of foreskin inflammation using cytokine/chemokine quantitation assayed by enzyme-linked immunosorbent assay (ELISA), defined as the presence of ≥ 3/7 inflammatory cytokines within the top quartile for that cytokine; 5) foreskin microbiota composition as characterized using 16S rRNA sequencing; and 6) foreskin tissue explant HIV susceptibility, based on p24 ELISA after ex vivo incubation with a primary HIV isolate.

### Study follow-up and procedures

**Fig. 1:**
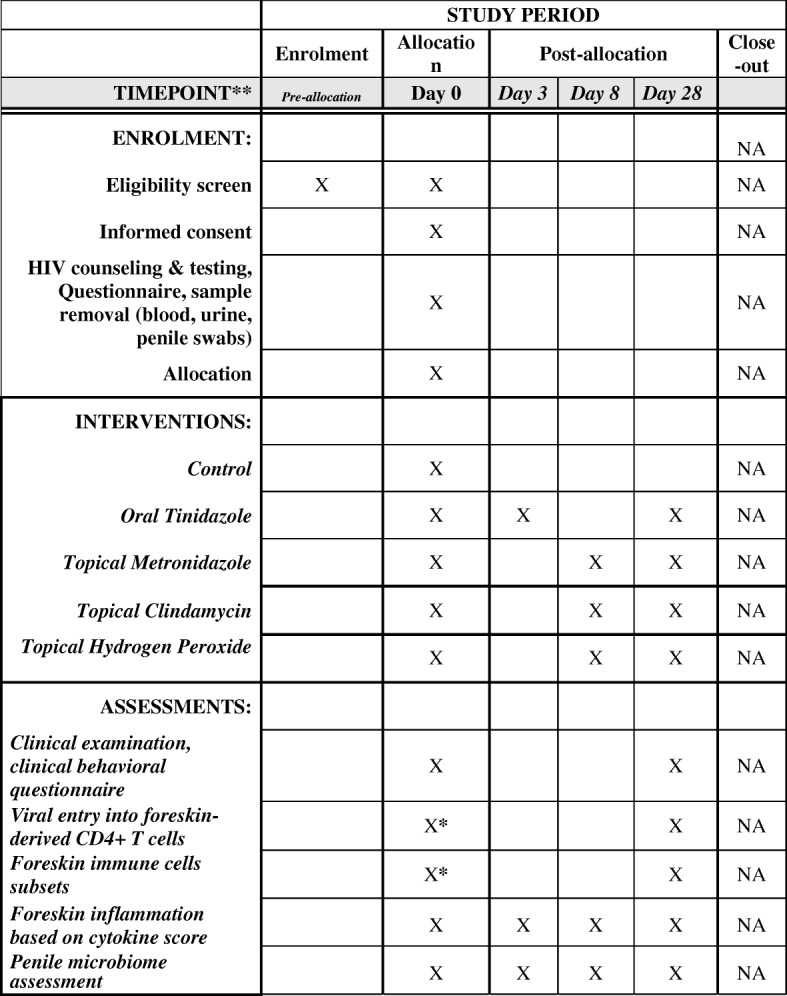
Schedule of enrolment, interventions, and assessments. *Only applicable for control/nonintervention groups samples. NA = not applicable

Potential study participants will be screened for eligibility (Table 1), confirmed to be generally healthy and without symptomatic genital infections, and then consented by study team clinical staff. Any person with sexually transmitted infection (STI) symptoms at screening, including genital ulceration, urethral discharge or dysuria, will be offered syndromic STI treatment as specified by the Uganda Ministry of Health guidelines and enrolment will be deferred; in addition, any participants found to have asymptomatic gonorrhea or chlamydia upon later polymerase chain reaction (PCR) urine screening will be provided with appropriate treatment. HIV counseling, condom provision, and rapid HIV testing are performed, a social-behavioral baseline questionnaire is administered, and the participant is randomized. Biological samples collected include peripheral venous blood, a first void urine specimen, and genital tract swab samples from the coronal sulcus, inner foreskin, and urethral meatus. All male participants will receive tetanus toxoid vaccination as recommended by the Uganda Ministry of Health guidelines prior to PC. Participants in group 2 have a post-treatment visit on day 3, and those in groups 3–5 on day 8. Men in intervention groups 3, 4, and 5, will be reminded about product use twice a week via a telephone call. After 4 weeks, men are interviewed about product use, tolerability, and sexual practices, provide a repeat blood sample and penile swabs, and undergo routine PC. The excised foreskin tissues are used for immune-based assays to assess the HIV susceptibility of foreskin-derived CD4^+^ T cells (see below). Participants are deemed lost to follow-up if they do not return within 3 days of a scheduled date/visit (see Fig. 1 for visit scheduling).

At the enrolment visit, female partners who opt to participate and are not actively menstruating will complete a questionnaire regarding genital symptoms and their menstrual cycle; they will self-collect undiluted female genital secretions using a cervicovaginal SoftCup, as well as two vaginal swabs, and provide a first void urine sample for STI testing and 6–8 ml of venous blood. Syndrome-based and PCR-based STI diagnosis and treatment are offered free of charge for all female participants. These study procedures are repeated at the time of her male partner’s PC procedure 4 weeks later.

## Biological sample processing

### Blood collection and processing

Peripheral venous blood is drawn in two Becton Dickinson Vacutainer^®^ acid citrate dextrose tubes. Plasma is isolated by centrifugation and stored at –80 °C for later confirmation of HIV and other tests including herpes simplex virus type 2 and syphilis.

### Genital swab collection and handling

Pre-moistened swabs are carefully applied to the coronal sulcus (two swabs), inner foreskin (two swabs) and urethral meatus (one swab) for men, while dry swabs are availed to women for two self-collected vaginal swabs. All swabs will be placed in clean microtubes/filter tubes and transported to the laboratory on ice. One unprocessed swab (from the inner foreskin and coronal sulcus) will be frozen as is at –80 °C while the others (from the urethra, inner foreskin and coronal sulcus) will be resuspended and stored at –80 °C in two aliquots each of 250 μL phosphate-buffered saline (PBS) mixed with a protease inhibitor and buffer.

### Cervical secretions collection

A flexible plastic cup (Softcup™, Instead) is self-inserted into the vagina and removed after 20 min. The secretions are then weighed and diluted six-fold in PBS and stored at –80 °C in 250 μL aliquots.

### Urine collection and handling

First-void urine is self-collected in a urine container and transported on ice to the laboratory where it is aliquoted into a PCR urine tube (Cobas^®^, Roche Molecular Systems, Inc.) prior to later *Neisseria gonorrhoeae* and *Chlamydia trachomatis* testing by PCR.

### Foreskin collection and processing

As previously described [23], the inner aspect of the foreskin is tagged during surgery with a suture, to aid subsequent tissue orientation, and is immediately transported at ambient temperature to the laboratory. Excess foreskin tissue and obvious blood clots are trimmed off, and tissue cut into 0.25-cm^2^ pieces that are added to 500 U/ml collagenase type 1 (Gibco #17100) and 42.5 U/ml deoxyribonuclease (Invitrogen) in RPMI 1640 media. Tissues are mechanically disrupted using scissors, followed by 30 min of enzymatic digestion at 37 °C and 900 rpm on a shaker (Eppendorf Thermo mixer, Hamburg, Germany). After digestion, cell suspensions are added to 3 ml cold fetal bovine serum (FBS) and filtered through a 100-μm cell strainer (BD Biosciences, Franklin Lakes, NJ, USA). Then 30 U/ml deoxyribonuclease is added to the filtrate, cells are washed and resuspended in R10 media (comprising RPMI 1640 media with 10% heat-inactivated FBS, 10 U/ml penicillin, 10 μg/ml streptomycin, 250 ng/ml amphotericin B, and 2 mM l-glutamine; Gibco, Invitrogen, Carlsbad, CA, USA) prior to overnight resting at 37 °C under 5% CO_2_. Following overnight rest, foreskin mononuclear cells are counted using trypan blue exclusion, and approximately 10 × 10^6^ mononuclear foreskin cells plated in 500 μl R10 media.

Cells are then stained for 30 min at 4 °C with labeled monoclonal antibodies specific for subsequent flow cytometry analysis. In addition, separate tissue samples from the inner and outer foreskin are cryopreserved in 10% dimethyl sulfoxide in FBS freezing media at –150 °C and in Cryomatrix media (ThermoScientific).

## Laboratory assays

### Pseudovirus cell entry assay

A β-lactamase-viral protein R (BlaM-Vpr)-containing HIV pseudovirus has been pseudotyped with an early transmitted, CCR5-tropic, Clade A envelope [21, 24]. Virion fusion with target cells delivers BlaM-Vpr into the cytosol, and cells are then loaded with the substrate CCF2-AM, a membrane-permeant form of the fluorescent molecule CCF that contains two fluorophores, 7-hydroxy-coumarin and fluorescein, linked by a β-lactam bond. In the absence of β-lactamase, excitation of 7-hydroxycoumarin at 409 nm leads to fluorescent resonance energy transfer (FRET) to fluorescein and green light emission at 520 nm. In infected cells, β-lactamase (delivered by BlaM-Vpr) cleaves the β-lactam bond of CCF2-AM, preventing FRET, and causing blue emission (447 nm) by 7-hydroxy-coumarin. Therefore, the ratio of blue to green emission is a measure of viral fusion and cytosolic entry of HIV. This assay quantifies the number of total and infected foreskin-derived CD4^+^ T cells and/or infected cell subsets without the need for in vitro cell activation, and this readout will constitute our primary endpoint. Assays in this open-label trial will be performed by unblinded investigators.

### Foreskin explant assay

Explants maintain the physiological tissue cellular and structural milieu, providing an extra way to study HIV susceptibility at the tissue level; however, they are generally semiquantitative and so constitute a secondary endpoint in our trial. Foreskin tissue strips (2 mm) are generated through gentle maceration and inoculated with HIV-1 in the presence of phytohemagglutinin (PHA), and IL-2 added to stimulate T cell activation and proliferation in the tissue. Control samples are also cultured without HIV-1 for each time interval. At three time points during the culturing process (days 3, 7, and 10), tissue culture media is removed and p24 ELISA used to measure viral replication in HIV-infected samples. When compared with controls without PHA/IL-2, PHA/IL-2^+^ cultures exhibit a faster and earlier increase in p24 expression during the culturing process, with maximum p24 expression observed at day 3.

### Immunofluorescence microscopy

Immunofluorescence microscopy will be used to objectively quantify immune cell populations in mucosal biopsies using image analysis software (Definiens) within a user-established depth (e.g., from the foreskin epidermis). This also permits quantitative assessment of epithelial integrity, which is important in studies of HIV susceptibility using foreskin tissues snap frozen into cryomolds in optimal cutting temperature (OCT) compound.

### Multiplex ELISA for cytokine/chemokine quantification

Cytokines will be quantified by chemiluminescent multiplex ELISA (MesoScale Discovery), an assay with optimal sensitivity and reproducibility for low abundance mucosal samples and validated for use in foreskin swabs [12–14]. Genital inflammation in the female genital tract (FGT) is predefined as the presence of ≥ 3/7 inflammatory cytokines within the top quartile for that cytokine.

### Penile and FGT microbiota characterization

The penile and FGT microbiota will be assessed using barcoded universal primers to amplify V4 hypervariable regions of 16S rRNA genes. Amplicons will be purified, quantified, and pooled prior to sequencing on the Illumina MiSeq platform using the 300 bp paired-end protocol [25]. Sequence read quality will be performed using a standardized bioinformatics pipeline implemented in accordance with the National Institutes of Health Human Microbiome Project standard operating procedures. Operational taxonomic units will be clustered against the Greengenes reference database and taxonomy assigned using –utax through USEARCH, a high-throughput sequence analysis tool that is implemented in QIIME [26] (an open-source microbiome bioinformatics platform). For each sample, vectors of phylotype proportions are clustered into community state types, by computing Jensen-Shannon distances between all pairs of community states and generating a hierarchical clustering using the Jensen-Shannon distance data and Ward linkage [14, 16, 27].

### Storage of biological samples

All samples will be stored at the UVRI-IAVI laboratory (Entebbe, Uganda) and/or the University of Toronto (Toronto, Canada). Samples will be stored for up to 10 years. Approvals from the University of Toronto and UVRI Research and Ethics Committees (RECs) will be sought prior to any additional testing on stored samples not listed in this protocol. Specific testing mentioned here and relevant to the study endpoints (immunofluorescence, cytokine/chemokine quantification, and microbiota profiling) will require the shipment of samples to Canada due to the lack of availability of the testing equipment within Uganda.

### Data collection and record keeping

All study forms/laboratory reports will be reviewed for accuracy and completion prior to data entry. Changes and corrections to the forms must be initialed and dated following the principles of Good Clinical Practice. The forms and source documentation or clinical notes will be kept in a secure location and archived for at least 10 years after study completion. Source documentation and other study-related records will be held at the UVRI-IAVI research data center with personal identifiers separated from participants’ files. Data quality and integrity will be ensured through the implementation of quality control procedures during database entry including double data entry, validation, and cleaning. Queries raised during data monitoring will be directed to study investigator(s) and study staff. A file will be held at a secure, central location at the UVRI-IAVI research center site for each participant containing all the source documents and written documentation on all queries raised and how they were addressed. Source documentation will be available for review to ensure that data collected and recorded in the database and case report forms are consistent with the contents of the source documents. Participant questionnaire and test result data will be coded and securely stored at the UVRI-IAVI premises research data unit. UVRI database access is limited to study staff and restricted by login and password; any database leaving the UVRI campus for analysis purposes, either electronically or in hard copy, will contain only unique study identifiers and will have been stripped of all personal identifiers (such as name, address, date of birth, contact details, and so on).

### Statistical analysis

The impact of antimicrobial agents on a clade A HIV pseudovirus entry into foreskin-derived CD4^+^ T cells (the primary endpoint) will be assessed by comparing each product group with the untreated control group, using a standard *t* test. The same statistical approach will be used for secondary immune endpoints listed above. The readout for explant HIV infection assays will be the median quantity of HIV p24 antigen produced, and the endpoint will be the percent change in infectivity compared with the vehicle control from the same foreskin, where percentage reduction = ((control − intervention)/control) × 100. A one-sample Wilcoxon-rank test will be used to determine significance (taken as *p* < 0.05). For participants in the intervention groups, the penile bacterial load, microbiota composition, and prepuce cytokine levels will be compared between baseline and on-product visits using a paired *t* test. In female participants, cervico-vaginal bacterial load, microbiota composition, and cytokine levels will be compared between the baseline and follow-up visit using a paired *t* test. In addition, for longitudinally assessed parameters, we will perform an analysis of covariance comparing each intervention group to the control group after adjustment for baseline differences. In case of reported product noncompliance, all participants with missing data will be analyzed according to the group to which they were randomized.

Differences in microbiota biodiversity will be assessed using two biodiversity metrics: diversity (D), calculated as D = Simpson’s diversity index, and evenness (E), calculated as E = D/S, where S is richness. Evenness reflects the dominance by many (i.e., high evenness) versus few (i.e., low evenness) operational taxonomic units (OTUs), whereas richness is a measurement of the total number of unique OTUs present [28]. Differences in overall microbiota composition will be assessed visually based on family-level log-transformed absolute abundance data using nMDS and Bray-Curtis distance; differences are assessed using permutational multivariate analysis of variance (PerMANOVA) based on the log-transformed abundance data [29–31].

### Sample size

Based on previously defined pseudovirus entry parameters into inner foreskin-derived CD4^+^ T cells [32], a sample size of 25 participants per group will provide statistical power of 80% to identify clinical approaches that reduce virus entry by ≥ 33% (based on a median virus entry of 15.1% into inner foreskin-derived CD4^+^ T cells), which is the efficacy threshold that we have deemed as sufficient to move a strategy forward.

### Reporting checklist

The SPIRIT reporting guidelines were used to compile the checklist for this protocol [33] (see Additional file 1).

## Dissemination of study results

Local dissemination of study results will be done in the form of oral presentations and printed pamphlets to research stakeholders, specifically UVRI-IAVI, the local Community Advisory Board (CAB) and the Entebbe General Hospital team. A final report with major study findings and a summary of drug adverse effects experienced by study participants will be submitted to both the UVRI-REC and the National Drug Authority (NDA) at the end of the study. Based on the scientific applicability, study findings will be presented at international conferences and published in peer-reviewed journals.

## Patient and public involvement

This clinical trial was informed by the suboptimal uptake of circumcision by men at risk of HIV in Uganda and more broadly across sub-Saharan Africa [8, 9]. Study participants were not directly involved in the study design and will not directly assess the effect of the interventions, but will play a role through peer referral of potential participants. The UVRI-IAVI CAB meets quarterly, and provided advice to the team regarding study design and recruitment; the CAB also serves as a conduit for community dissemination of results.

## Discussion

The steep decline in global HIV incidence observed in the 1990s and early 2000s has recently plateaued [34], indicating a critical need to refocus HIV prevention efforts and develop community-appropriate prevention tools. Antiretroviral-based strategies, including the provision of PrEP to high-risk individuals and early diagnosis and treatment in HIV-positive individuals, are highly effective [35–37], but barriers such as cost and access to care pose a major challenge to their roll out in many regions of sub-Saharan Africa [38–43]. Importantly, the global financing of HIV treatment programs has declined over the past few years and many governments have not shown solid commitment to patch this gap [44–46]. While PC is a one-time intervention with lasting efficacy, many eligible men are unwilling to undergo this procedure [8, 9]. Therefore, there is a need for alternative tools to reduce HIV risk in uncircumcised men from endemic countries. Based on recent studies linking the foreskin microbiota with local penile inflammation, increased HIV target cell density, and enhanced HIV acquisition risk, this clinical study will assess the impact of commonly used antibacterial agents on foreskin HIV susceptibility, immunology, and microbiota in Ugandan men.

The mechanism that links the foreskin microbiota with subsequent HIV risk is likely to include enhanced tissue recruitment of HIV-susceptible CD4^+^ T cells, mediated by chemoattractants such as IL-8 that are released by epithelial cells after the recognition of pathogen-associated molecular patterns by pattern-recognition receptors such as Toll-like receptors [47, 48], although inflammatory cytokines may also directly reduce epithelial integrity [49]. While circumcision also directly removes HIV-susceptible tissue, a role for the microbiota is further suggested by the link between the microbiota and HIV acquisition among men who remain uncircumcised [14] as well as studies in Papua New Guinea where HIV risk is reduced among men who have undergone a dorsal slit technique that maintains exactly the same tissue volume but induces an aerobic environment [50, 51]. The latter studies imply that it is the composition of the preputial microbiota and associated inflammation, rather than the presence of foreskin tissue itself, that are key determinants of HIV acquisition risk. Taken together, these observations suggest that, while penile HIV susceptibility is a multifaceted process, immune susceptibility to HIV is shaped by the penile microbiota.

While the penile microbiota may be a central determinant of HIV risk, it is not known whether and/or in what way common antimicrobials will alter the composition of the foreskin microbiota. In addition, it is an important point of principle to demonstrate that such changes, once induced, can alter local tissue immunology and HIV susceptibility. Therefore, this randomized, open-label clinical trial aims to establish the impact of four widely available, cheap, and well-tolerated antimicrobials on foreskin immunology, HIV susceptibility, and microbiota. While not intuitively a major concern, a prespecified subanalysis will assess the safety and immune/microbial effects of male penile antimicrobial use in the genital tract of long-term female partners, if applicable. One strength of the proposed approach is that the antimicrobials being investigated are generally low-cost, already licensed for use in Uganda, and have demonstrated efficacy against bacterial vaginosis, a condition that is mediated by several bacterial species also linked with penile inflammation and HIV susceptibility [14, 16]. However, should any product be demonstrated to favorably alter the penile microbiota and/or HIV susceptibility, important future questions for study would include the time course and duration of these changes, longer-term participant acceptability of product application, and the long-term potential for side effects or antibiotic resistance.

Our primary endpoint is the impact of each clinical intervention on HIV entry into foreskin-derived CD4^+^ T cells, compared with the no-intervention group. In addition to these four primary endpoints, we will be assessing multiple secondary endpoints, both immunological and microbiological, meaning that some statistically significant associations found may be due to chance alone. Our sample size does not permit us to rigorously control for multiple comparisons. However, since no prior studies have assessed the ability of a microbiome-based intervention to reduce penile HIV susceptibility, all trial outcomes other than tolerability will be primarily treated as hypothesis generating, with the goal of identifying 1–2 clinical approaches to take forward into more rigorous clinical testing.

In conclusion, observational studies suggest that penile HIV susceptibility in uncircumcised men is linked to inflammation caused by foreskin anaerobes. Since antibiotics targeting similar bacteria in the context of bacterial vaginosis are cheap, safe, and widely available, our randomized, open-label clinical trial will assess the impact of four antimicrobial approaches on the foreskin microbiota and ex vivo HIV susceptibility.

## Trial status and summary

Study participant enrollment began on 7 December 2017 using protocol version 1.2, dated17 July 2017. Expected completion of enrollment is January 2019.

## Additional file

Additional file 1: SPIRIT 2013 checklist: recommended items to address in a clinical trial protocol and related documents. (DOC 123 kb)

## Data Availability

Following completion of the trial, expected in 2019, the trial protocol and select de-identified trial data (including texts, tables, figures) used for analysis and publication will be available upon reasonable request from the corresponding author.

## Abbreviations

BlaM-Vpr: β-lactamase viral protein R
CAB: Community Advisory Board
ELISA: Enzyme-linked immunosorbent assay
FBS: Fetal bovine serum
FGT: Female genital tract
IAVI: International Aids Vaccine Initiative
IL: Interleukin
NDA: National Drug Authority
PBS: Phosphate-buffered saline
PC: Penile circumcision
PCR: Polymerase chain reaction
PHA: Phytohemagglutinin
PrEP: Pre-exposure prophylaxis
REC: Research and Ethics Committee
STI: Sexually transmitted infection
UVRI: Uganda Virus Research Institute

## References

[1] Baggaley R, Doherty M, Ball A, Ford N, Hirnschall G (2015). The strategic use of antiretrovirals to prevent HIV infection: a converging agenda. Clin Infect Dis.

[2] Rodger AJ, Cambiano V, Bruun T, Vernazza P, Collins S, Degen O, Corbelli GM, Estrada V, Geretti AM, Beloukas A, et al. Risk of HIV transmission through condomless sex in serodifferent gay couples with the HIV-positive partner taking suppressive antiretroviral therapy (PARTNER): final results of a multicentre, prospective, observational study. Lancet. 2019;393(10189):2428–38. 10.1016/S0140-6736(19)30418-0 PMC6584382 31056293

[3] UNAIDS (2018). Global HIV & AIDS statistics — 2018 fact sheet.

[4] TenoRes Study G (2016). Global epidemiology of drug resistance after failure of WHO recommended first-line regimens for adult HIV-1 infection: a multicentre retrospective cohort study. Lancet Infect Dis.

[5] Auvert B, Taljaard D, Lagarde E, Sobngwi-Tambekou J, Sitta R, Puren A (2005). Randomized, controlled intervention trial of male circumcision for reduction of HIV infection risk: the ANRS 1265 Trial. PLoS Med.

[6] Bailey RC, Moses S, Parker CB, Agot K, Maclean I, Krieger JN, Williams CF, Campbell RT, Ndinya-Achola JO (2007). Male circumcision for HIV prevention in young men in Kisumu, Kenya: a randomised controlled trial. Lancet.

[7] Gray RH, Kigozi G, Serwadda D, Makumbi F, Watya S, Nalugoda F, Kiwanuka N, Moulton LH, Chaudhary MA, Chen MZ (2007). Male circumcision for HIV prevention in men in Rakai, Uganda: a randomised trial. Lancet.

[8] Gray RH, Wawer MJ, Kigozi G (2013). Programme science research on medical male circumcision scale-up in sub-Saharan Africa. Sex Transm Infect.

[9] Sgaier SK, Reed JB, Thomas A, Njeuhmeli E (2014). Achieving the HIV prevention impact of voluntary medical male circumcision: lessons and challenges for managing programs. PLoS Med.

[10] Kigozi G, Wawer M, Ssettuba A, Kagaayi J, Nalugoda F, Watya S, Mangen FW, Kiwanuka N, Bacon MC, Lutalo T (2009). Foreskin surface area and HIV acquisition in Rakai, Uganda (size matters). AIDS.

[11] Prodger JL, Gray R, Kigozi G, Nalugoda F, Galiwango R, Hirbod T, Wawer M, Hofer SO, Sewankambo N, Serwadda D, Kaul R (2012). Foreskin T-cell subsets differ substantially from blood with respect to HIV co-receptor expression, inflammatory profile, and memory status. Mucosal Immunol.

[12] Prodger JL, Hirbod T, Kigozi G, Nalugoda F, Reynolds SJ, Galiwango R, Shahabi K, Serwadda D, Wawer MJ, Gray RH (2014). Immune correlates of HIV exposure without infection in foreskins of men from Rakai, Uganda. Mucosal Immunol.

[13] Prodger JL, Gray RH, Shannon B, Shahabi K, Kong X, Grabowski K, Kigozi G, Nalugoda F, Serwadda D, Wawer MJ (2016). Chemokine levels in the penile coronal sulcus correlate with HIV-1 acquisition and are reduced by male circumcision in Rakai, Uganda. PLoS Pathog.

[14] Liu CM, Prodger JL, Tobian AAR, Abraham AG, Kigozi G, Hungate BA, Aziz M, Nalugoda F, Sariya S, Serwadda D, et al. Penile anaerobic dysbiosis as a risk factor for HIV infection. MBio. 2017;8. 10.1128/mBio.00996-17. 10.1128/mBio.00996-17 PMC5527312 28743816

[15] Esra RT, Olivier AJ, Passmore JA, Jaspan HB, Harryparsad R, Gray CM (2016). Does HIV exploit the inflammatory milieu of the male genital tract for successful infection?. Front Immunol.

[16] Liu CM, Hungate BA, Tobian AA, Ravel J, Prodger JL, Serwadda D, Kigozi G, Galiwango RM, Nalugoda F, Keim P (2015). Penile microbiota and female partner bacterial vaginosis in Rakai, Uganda. MBio.

[17] Armstrong NR, Wilson JD (2010). Tinidazole in the treatment of bacterial vaginosis. Int J Women’s Health.

[18] Menard JP (2011). Antibacterial treatment of bacterial vaginosis: current and emerging therapies. Int J Women’s Health.

[19] Jhingta P, Bhardwaj A, Sharma D, Kumar N, Bhardwaj VK, Vaid S (2013). Effect of hydrogen peroxide mouthwash as an adjunct to chlorhexidine on stains and plaque. J Indian Soc Periodontol.

[20] Rashed HT (2016). Evaluation of the effect of hydrogen peroxide as a mouthwash in comparison with chlorhexidine in chronic periodontitis patients: a clinical study. J Int Soc Prev Community Dent.

[21] Joag VR, McKinnon LR, Liu J, Kidane ST, Yudin MH, Nyanga B, Kimwaki S, Besel KE, Obila JO, Huibner S (2016). Identification of preferential CD4+ T-cell targets for HIV infection in the cervix. Mucosal Immunol.

[22] Cavrois M, De Noronha C, Greene WC (2002). A sensitive and specific enzyme-based assay detecting HIV-1 virion fusion in primary T lymphocytes. Nat Biotechnol.

[23] Galiwango RMYS, Prodger JL (2016). HIV susceptibility in CD4+ T cells derived from the Inner versus outer foreskin. HIV Research for prevention 2016: AIDS vaccine, microbicide and ARV-based prevention science.

[24] Joag V, Sivro A, Yende-Zuma N, Imam H, Samsunder N, Abdool Karim Q, Abdool Karim S, McKinnon L, Kaul R (2018). Ex vivo HIV entry into blood CD4+ T cells does not predict heterosexual HIV acquisition in women. PLoS One.

[25] Fadrosh DW, Ma B, Gajer P, Sengamalay N, Ott S, Brotman RM, Ravel J (2014). An improved dual-indexing approach for multiplexed 16S rRNA gene sequencing on the Illumina MiSeq platform. Microbiome.

[26] Caporaso JG, Kuczynski J, Stombaugh J, Bittinger K, Bushman FD, Costello EK, Fierer N, Pena AG, Goodrich JK, Gordon JI (2010). QIIME allows analysis of high-throughput community sequencing data. Nat Methods.

[27] Liu CM, Aziz M, Kachur S, Hsueh PR, Huang YT, Keim P, Price LB (2012). BactQuant: an enhanced broad-coverage bacterial quantitative real-time PCR assay. BMC Microbiol.

[28] Simpson EH (1949). Measurement of diversity. Nature.

[29] Oksanen J, Blanchet FG, Kindt R, Legendre P, Minchin PR, O'Hara RB, Simpson GL, Solymos P, Stevens HH, Wagner H (2011). Vegan: community ecology package. R Package Version 1.

[30] Kindt RC (2005). Tree diversity analysis. A manual and software for common statistical methods for ecological and biodiversity studies.

[31] Goslee SC, Urban DL (2007). The ecodist package for dissimilarity-based analysis of ecological data. J Stat Softw.

[32] Galiwango RM, Yegorov S, Joag V, Prodger J, Shahabi K, Huibner S, Muyanja E, Kabuubi BR, Namuniina A, Nalutaaya A (2019). Characterization of CD4(+) T cell subsets and HIV susceptibility in the inner and outer foreskin of Ugandan men. Am J Reprod Immunol.

[33] Chan AW, Tetzlaff JM, Altman DG, Laupacis A, Gotzsche PC, Krleza-Jeric K, Hrobjartsson A, Mann H, Dickersin K, Berlin JA (2013). SPIRIT 2013 statement: defining standard protocol items for clinical trials. Ann Intern Med.

[34] Collaborators GH (2016). Estimates of global, regional, and national incidence, prevalence, and mortality of HIV, 1980–2015: the Global Burden of Disease Study 2015. Lancet HIV.

[35] Baeten JM, Donnell D, Ndase P, Mugo NR, Campbell JD, Wangisi J, Tappero JW, Bukusi EA, Cohen CR, Katabira E (2012). Antiretroviral prophylaxis for HIV prevention in heterosexual men and women. N Engl J Med.

[36] Cohen MS, Chen YQ, McCauley M, Gamble T, Hosseinipour MC, Kumarasamy N, Hakim JG, Kumwenda J, Grinsztejn B, Pilotto JH (2011). Prevention of HIV-1 infection with early antiretroviral therapy. N Engl J Med.

[37] Grinsztejn B, Hosseinipour MC, Ribaudo HJ, Swindells S, Eron J, Chen YQ, Wang L, Ou SS, Anderson M, McCauley M (2014). Effects of early versus delayed initiation of antiretroviral treatment on clinical outcomes of HIV-1 infection: results from the phase 3 HPTN 052 randomised controlled trial. Lancet Infect Dis.

[38] Gils T, Bossard C, Verdonck K, Owiti P, Casteels I, Mashako M, Van Cutsem G, Ellman T (2018). Stockouts of HIV commodities in public health facilities in Kinshasa: barriers to end HIV. PLoS One.

[39] Harper KN (2016). Does the shortage of antiretroviral drugs in Uganda signal a trend?. AIDS.

[40] Mori AT, Owenya J (2014). Stock-outs of antiretroviral drugs and coping strategies used to prevent changes in treatment regimens in Kinondoni District, Tanzania: a cross-sectional study. J Pharm Policy Pract.

[41] Moriarty K, Genberg B, Norman B, Reece R (2018). The effect of antiretroviral stock-outs on medication adherence among patients living with HIV in Ghana: a qualitative study. J Assoc Nurses AIDS Care.

[42] Zakumumpa H, Dube N, Damian RS, Rutebemberwa E (2018). Understanding the dynamic interactions driving the sustainability of ART scale-up implementation in Uganda. Glob Health Res Policy.

[43] Ayieko J, Brown L, Anthierens S, Van Rie A, Getahun M, Charlebois ED, Petersen ML, Clark TD, Kamya MR, Cohen CR (2018). “Hurdles on the path to 90-90-90 and beyond”: qualitative analysis of barriers to engagement in HIV care among individuals in rural East Africa in the context of test-and-treat. PLoS One.

[44] Kakaire T, Schlech W, Coutinho A, Brough R, Parkes-Ratanshi R (2016). The future of financing for HIV services in Uganda and the wider sub-Saharan Africa region: should we ask patients to contribute to the cost of their care?. BMC Public Health.

[45] Resch S, Ryckman T, Hecht R (2015). Funding AIDS programmes in the era of shared responsibility: an analysis of domestic spending in 12 low-income and middle-income countries. Lancet Glob Health.

[46] Schneider MT, Birger M, Haakenstad A, Singh L, Hamavid H, Chapin A, Murray CJ, Dieleman JL (2016). Tracking development assistance for HIV/AIDS: the international response to a global epidemic. AIDS.

[47] Prodger JL, Kaul R (2017). The biology of how circumcision reduces HIV susceptibility: broader implications for the prevention field. AIDS Res Ther.

[48] de Jong MA, de Witte L, Oudhoff MJ, Gringhuis SI, Gallay P, Geijtenbeek TB (2008). TNF-alpha and TLR agonists increase susceptibility to HIV-1 transmission by human Langerhans cells ex vivo. J Clin Invest.

[49] Nazli A, Chan O, Dobson-Belaire WN, Ouellet M, Tremblay MJ, Gray-Owen SD, Arsenault AL, Kaushic C (2010). Exposure to HIV-1 directly impairs mucosal epithelial barrier integrity allowing microbial translocation. PLoS Pathog.

[50] MacLaren DJ, McBride WJ, Kelly GC, Muller R, Tommbe R, Kaldor JM, Vallely AJ (2015). HIV prevalence is strongly associated with geographical variations in male circumcision and foreskin cutting in Papua New Guinea: an ecological study. Sex Transm Infect.

[51] Vallely AJ, MacLaren D, David M, Toliman P, Kelly-Hanku A, Toto B, Tommbe R, Kombati Z, Kaima P, Browne K (2017). Dorsal longitudinal foreskin cut is associated with reduced risk of HIV, syphilis and genital herpes in men: a cross-sectional study in Papua New Guinea. J Int AIDS Soc.

